# α-Synuclein induced cholesterol lowering increases tonic and reduces depolarization-evoked synaptic vesicle recycling and glutamate release

**DOI:** 10.1038/s41531-022-00334-7

**Published:** 2022-06-07

**Authors:** Vesna Lazarevic, Yunting Yang, Wojciech Paslawski, Per Svenningsson

**Affiliations:** Department of Clinical Neuroscience, Karolinska Institute, Stockholm, Sweden

**Keywords:** Parkinson's disease, Neurotransmitters

## Abstract

α-Synuclein (α-syn) is a key molecule linked to Parkinson’s disease pathology. Physiologically, the monomeric α-syn in the presynaptic termini is involved in regulation of neurotransmission, but the pathophysiology of extracellular monomeric α-syn is still unknown. Utilizing both in vivo and in vitro approaches, we investigated how extracellular α-syn impact presynaptic structure and function. Our data revealed that treatment with exogenous α-syn leads to increased tonic and decreased depolarization-evoked synaptic vesicle (SV) recycling and glutamate release. This was associated with mobilization of molecularly distinct SV pools and reorganization of active zone components. Our study also showed that exogenous α-syn impaired neuronal cholesterol level and that the cholesterol binding domain of α-syn was sufficient to exert the same presynaptic phenotype as the full-length protein. The present study sheds new light on physiological functions of extracellular α-syn in overall maintenance of presynaptic activity that involves the reorganization of both presynaptic compartment and cholesterol-rich plasma membrane domains.

## Introduction

α-Synuclein (α-syn) is involved in the etiology of Parkinson’s disease (PD) and several other neurodegenerative disorders commonly known as synucleinopathies^1,2^. One of the major molecular mechanisms underlying these pathological conditions is misfolding and abnormal aggregation of α-syn that eventually leads to synaptic dysfunction and neuronal cell death. α-Syn was also determined as the key component of neuropathological inclusions, Lewy bodies, and Lewy neurites, the main pathological hallmarks of synucleinopathies, including PD and dementia with Lewy bodies (DLB)^3,4^.

Notwithstanding important progress towards understanding the physiology of α-syn, major questions regarding its effect on synapse function and how that may lead to pathology of PD and other synucleinopathies remain unresolved. Being mainly presynaptically expressed, this protein has been involved in the regulation and maintenance of synaptic vesicle (SV) recycling and neurotransmitter release. Numerous in vitro and in vivo studies associated with either α-syn loss or gain of function, revealed its involvement in different steps of SV trafficking from reserved to readily releasable pool (RRP), including docking, priming, and fusion of release-competent SVs as well as SVs reclustering after neurotransmitter release^5,6^. Of particular importance are studies showing that α-syn directly binds to SNARE protein synaptobrevin-2/VAMP2 promoting SNARE-complex assembly^7^ and facilitating SNARE-dependent SVs docking^8,9^.

The majority of α-syn is intracellular, but numerous studies convincingly support the existence of extracellular forms of this protein as well. α-Syn is shown to be released from neurons under both physiological and pathological conditions^10–16^. Moreover, it has been shown that the secretion of α-syn is dependent on intrinsic neuronal activity^17^. Due to its ability to propagate from cell to cell and from one brain region to another in a “prion-like” manner^18^, the current research in the field of α-syn is shifting from studies of intracellular to extracellular forms of the protein. There are a number of recent studies describing the effect of extracellular α-syn binding to a variety of cell surface receptors expressed on both neuronal and glial cells. These effects range from Ca^2+^ dysregulation and synaptic dysfunction to neurodegeneration and cognitive impairments^19^.

One of the most important aspects of α-syn (patho)physiology is its interaction with lipid membranes^20^, among others with lipid microdomains resistant to low-temperature detergent solubilization, so-called, lipid rafts. The association of α-syn with lipid rafts plays an important role in the physiological activity of the protein. Disruption of this association redistributes α-syn away from the synapse and impairs its cellular function^21^. On the other hand, exogenously applied α-syn may exert impairment of lipid raft composition that alters downstream signaling pathways and synaptic activity^22,23^. A recent study, using advanced correlative light and electron microscopy on human tissue, reported the presence of lipid membrane fragments in close proximity to α-syn in Lewy bodies, making the association of α-syn and lipids even more important for the understanding Lewy pathology and PD pathogenesis^24^.

It has been discovered that α-syn contains a high-affinity cholesterol-binding motif in the 67–78 aa region^25^ and this interaction was shown to be essential for the formation of cytotoxic ‘amyloid pores’^26^. Cholesterol itself is known to have a crucial role in regulating all steps of neurotransmission from action potential (AP) conduction to neurotransmitter release and postsynaptic receptor activation^27^. The finding that extracellular α-syn reduces the amount of membrane cholesterol^28^, led to the hypothesis that α-syn action on synapse (patho)physiology involves the reorganization of plasma membrane microdomains, specifically the ones enriched in cholesterol, that consequently may alter neurotransmission.

In the present study, we provide novel evidence for the physiological role of extracellular α-syn in regulating presynaptic structure and function that is governed by alteration of the membrane cholesterol content. Namely, our data revealed that exogenous α-syn impairs cholesterol level that subsequently triggers a specific signal which, in turn, mobilizes molecularly distinct SV populations within presynaptic terminals, leading to increased tonic and decreased depolarization-evoked SV recycling and glutamate release. We also showed that the effect of exogenous α-syn on presynaptic activity involves the cholesterol efflux protein, ABCA1, endogenous α-syn, and phosphorylation/dephosphorylation of presynaptic scaffolding protein, synapsin.

## Results

### Extracellular α-synuclein alters glutamate release and synaptic vesicle recycling

A number of previous studies suggested that the application of various exogenous α-syn proteoforms has both positive and negative effects on neuronal activity, inducing either increase^29,30^ or decrease of neurotransmitter release^31–33^. In the present study, we aimed to enlighten the functional role of exogenous α-syn, primarily focusing on its presynaptic effects. Combining both in vivo and in vitro approaches, we investigated how the application of exogenous α-syn regulates the presynaptic neurotransmitter release, SVs recycling, and active zone composition.

**Fig. 1:**
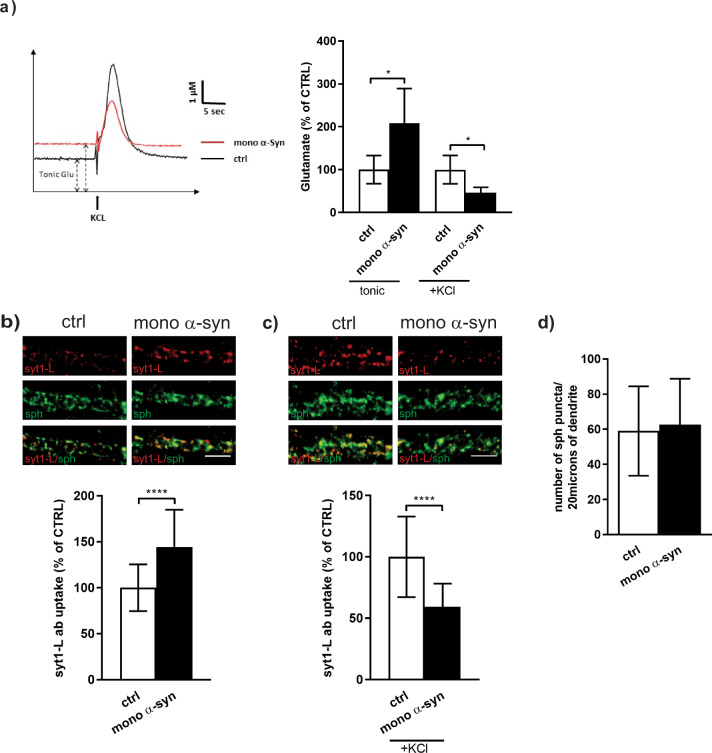
Exogenous α-synuclein alters glutamate release and synaptic vesicle recycling. **a** Graphical traces and quantification of tonic and depolarization-evoked (KCl) glutamate release measured in the prelimbic region of prefrontal cortex in mice upon local application of saline (ctrl; *N* = 4 animals) or 500 nM of recombinant monomeric α-syn (mono α-syn; *N* = 4 animals). Statistical significance was assessed using Student’s *t* test. *p < 0.05. **b** Representative images and corresponding quantification of network-activity-driven syt1L-ab uptake (red) in primary cortical neurons treated with control solution (ctrl; *N* = 88 cells) or 500 nM mono α-syn (*N* = 69 cells) for 90 min. Synaptophysin (sph; green) was used as a synaptic marker. Data are obtained from six independent experiments. Statistical significance was evaluated using Mann–Whitney *U*-test. ****p < 0.0001. **c** Representative images and corresponding quantification of KCl-depolarization-induced syt1L-ab uptake (red) in primary cortical neurons treated with control solution (ctrl; *N* = 43 cells) or 500 nM mono α-syn (*N* = 42 cells) for 90 min. Synaptophysin (sph; green) was used as a synaptic marker. Data are obtained from four independent experiments. Statistical significance was evaluated using Mann–Whitney *U*-test. ****p < 0.0001. **d** Bar graph denotes the number of sph-positive puncta along 20 µm of proximal dendrite in control (*N* = 72) and α-syn-treated cells (500 nM; 90 min; *N* = 59). Mann–Whitney *U*-test. *p* = 0.512. Data originate from six independent experiments. **b**–**d** All bars denote intensity values normalized to the mean intensity value in the control group ± SD per each experimental setup. All treatments were done at DIV 21–23. Scale bar 5 µm.

Enzyme-based microelectrode array coupled with amperometric recordings (using Fast Analytic Sensing Technology (FAST) methodology) revealed that acute local application of recombinant monomeric α-syn (500 nM) into the prelimbic region of the prefrontal cortex significantly enhanced tonic glutamate release and concomitantly caused more than twofold decrease in the KCL-evoked glutamate release (Fig. 1a). Taking into account that the FAST technology utilizes high temporal and spatial resolution that allows to estimate presynaptically released glutamate^34^, we further wanted to decipher how exogenous α-syn affects presynaptic function, including SV recycling and SV pools organization and the dynamics at the individual nerve terminals. Thus, we employed an in vitro approach using synaptotagmin 1 antibody uptake assay (syt1L-ab uptake) on primary cortical neurons. This method is based on fluorophore-coupled syt1 lumenal domain antibody that is internalized into the lumen of SVs undergoing exo-endocytosis, so the uptaken fluorescent signal closely reflects the level of SVs recycling at a given synapse. As shown in Fig. 1b, treatment of primary neurons with 500 nM monomeric α-syn was associated with augmentation of network activity driven (tonic) syt1L-ab uptake. On the other hand, KCl-induced depolarization (50 mM, 4 min) significantly attenuated the presynaptic response to exogenous α-syn (Fig. 1c). This effect was shown to be concentration dependent with no effect of lower nM range (50 nM) and the same effect with higher 5 µM treatment (Supplementary Fig. 1a, b). The total number of synaptic contacts, defined as synaptophysin positive puncta, remained unchanged (Fig. 1d). The same phenotype, increased tonic and reduced KCl-evoked SV recycling, was also observed in cells exposed to 500 nM of oligomeric or aggregated (α-syn preform fibrils, PFF) forms of α-syn (Supplementary Fig. 1c, d). In contrast to treatment with α-syn, when primary neurons were exposed for 90 min to 500 nM of β-synuclein, we did not observe any effect on SV recycling (Supplementary Fig. 1e) suggesting specificity of α-syn induced effect.

### α-Synuclein modulates presynaptic activity by mobilizing molecularly distinct SV populations

Several studies proposed that SVs involved in spontaneous and evoked neurotransmission originate from distinct pools of vesicles with limited mutual interference^35^. While depolarization-evoked release requires classical SNARE-complex and the presence of active-zone (AZ) proteins involved in SVs priming and docking^36^, spontaneous SV depletion is selectively maintained by non-canonical SNAREs, including Vti1a and VAMP7^37^.

**Fig. 2:**
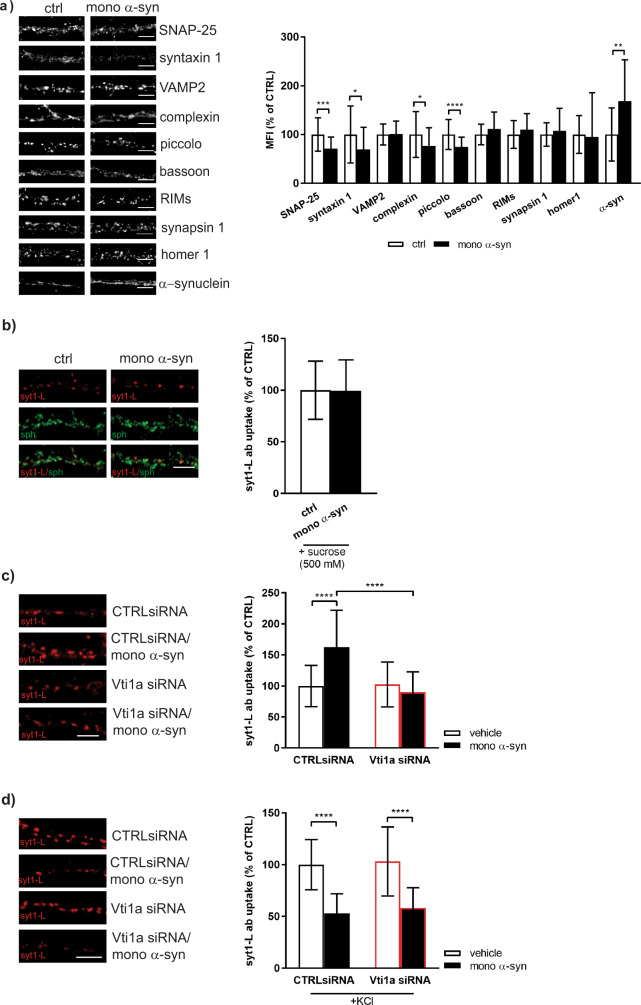
Exogenous α-synuclein modulation of presynaptic activity involves canonical and non-canonical SNAREs. **a** Representative images and bar graph displaying mean fluorescence intensity of selected pre-and postsynaptic proteins in control (ctrl) and neurons treated with monomeric α-syn (500 nM, 90 min). Data are obtained from 3 to 4 independent experiments. All treatments were done at DIV 21–23. Within each experiment, the intensity values of treated cells were normalized to the mean intensity value of the control group and expressed as mean ± SD. Statistical significance was assessed using Student’s *t* test (SNAP-25, synatxin1, VAMP2, bassoon, RIMs) or Mann–Whitney *U*-test (complexin, piccolo, synapsin 1, homer 1, α-syn). *p < 0.05 **p < 0.01 ***p < 0.001 ****p < 0.0001. **b** Representative images and corresponding quantification of the syt1L-ab uptake (red) in the presence of 500 nM sucrose. Primary cortical neurons were either treated with control solution (ctrl; *N* = 43) or with 500 nM of recombinant monomeric α-syn for 90 min (*N* = 41). Synaptophysin (sph; green) was used as a synaptic marker. Data are obtained from four independent experiments. Treatments were done at DIV 21–23. Within each experiment, values in treated cells are normalized to the mean value of the control group and expressed as mean ± SD. Mann–Whitney *U*-test. *p* = 0.6887. Scale bar 5 µm. **c**, **d** Primary cortical neurons (DIV 18) were exposed to CTRL siRNA or Vti1a siRNA 96 h prior 90 min treatment with control solution or 500 nM of monomeric α-syn. **c** Network-activity-driven presynaptic activity assessed by syt1L-ab uptake assay. Number of analyzed cells: CTRL siRNA (*N* = 31); CTRL siRNA/ mono α-syn (*N* = 35); Vti1a siRNA (*N* = 35); Vti1a siRNA/mono α-syn (*N* = 40). Data originate from three independent experiments. Within each experiment, the intensity values of treated cells were normalized to the mean intensity value of the control group (CTRL siRNA) and expressed as mean ± SD. Statistical significance was assessed using two-way ANOVA followed by Tukey’s post hoc test. Interaction *F*(1,137) = 28.2, *p* < 0.0001; Vti1a siRNA *F* = 24.53, *p* < 0.0001; mono α-syn *F* = 12.7, *p* < 0.0001. ****p < 0.0001. Scale bar 5 µm. **d** KCl-evoked syt1L-ab uptake. Number of analyzed cells: CTRL siRNA (*N* = 24); CTRL siRNA/mono α-syn (*N* = 33); Vti1a siRNA (*N* = 27); Vti1a siRNA/mono α-syn (*N* = 32). Data originate from three independent experiments. Within each experiment, the intensity values of treated cells were normalized to the mean intensity value of the control group (CTRL siRNA) and expressed as mean ± SD. Statistical significance was assessed using two-way ANOVA followed by Tukey’s post hoc test. Interaction *F*(1,112) = 0.05, *p* = 0.8255; Vti1a siRNA *F* = 0.84, *p* = 0.3624; mono α-syn *F* = 102.8, *p* < 0.0001. ****p < 0.0001. Scale bar 5 µm.

We showed here that treatment of primary neurons with α-syn impaired the level of certain SNAREs and SNARE-binding molecules per individual synaptic spot. As shown in Fig. 2a, α-syn treatment reduced the amount of t-SNAREs, SNAP25 and syntaxin 1, while no change of VAMP2/synaptobrevin was observed. Also, the same treatment lowered the synaptic level of SNARE-associated molecule, complexin, and AZ protein, piccolo. Therefore, the reduction of KCl-evoked presynaptic activity upon α-syn treatment might be explained by the impairment of SNARE-complex assembly. Other presynaptic proteins, known to be involved in SV recycling, were not significantly disturbed by extracellular α-syn (bassoon, RIMs, synapsin 1). Interestingly, treatment with exogenous α-syn led also to significant upregulation of endogenous α-syn levels within the individual synapse.

In order to determine how these structural changes within individual AZs impact SV pool size, we performed syt1L-ab uptake assay in control and α-syn treated cells in the presence of hypertonic solution (500 mM sucrose), a condition used to selectively deplete the RRP of SVs^38^. As shown in Fig. 2b, RRP was not affected by the exogenous α-syn. Additionally, the size of SV pools and their dynamics were also evaluated using a live imaging technique coupled with electrical stimulation. All recycling-competent vesicles of control and cells exposed to oligomeric α-syn were pre-labeled with syt1 lumenal domain antibody tagged with pH-sensitive fluorophore cypHer5E (syt1L-cypHer5E) and subsequently their exocytosis was evoked by field stimulation (40 AP at 20 Hz was used to release vesicles from the RRP and 200 AP at 20 Hz was applied to release vesicles from recycling pool (RP)). As shown in Supplementary Fig. 2, oligomeric α-syn treatment did not affect the size of RRP but significantly reduced the total RP.

To unravel the molecular basis for the fusion of SV subsets that drive tonic presynaptic activity in cells treated with α-syn, we used a siRNA approach to knock down the expression of Vti1a protein. The efficacy of Vti1a siRNA was confirmed by both ICC and WB (Supplementary Fig. 3a, b). In Vti1a KD cells AP-dependent presynaptic activity, depicted as syt1L-ab uptake signal in the presence of tetrodotoxin (TTX), was significantly reduced if compared to control cells (Supplementary Fig. 3c). Interestingly, the network-activity driven syt1L-ab uptake was not affected by Vti1a KD in control cells, but Vti1a seems to be critical for α-syn induced presynaptic potentiation (Fig. 2c). At the same time, depolarization-evoked SV recycling in both control and α-syn-treated cells was shown to be resistant to Vti1a KD (Fig. 2d).

Taken together, these results suggested that exogenous α-syn differently modulates the two modes of neurotransmission interfering with the size and dynamic organization of SV pools within presynaptic boutons. Depending on intrinsic neuronal activity α-syn may trigger a specific signal that mobilizes molecularly distinct SV populations within the presynaptic terminal driving tonic or evoked glutamate release.

### α-Synuclein induced presynaptic potentiation requires a direct Ca^2+^ influx through N-type VGCCs

To investigate whether exogenously applied α-syn targets all over synaptic contacts or its effect is restricted to a subset of synapses, we selectively measured syt1L-ab uptake in synapses positive for either VGLUT1 (excitatory synapses) or VGAT (inhibitory synapses). Our data revealed that α-syn potentiates the tonic activity in both types of synapses (Supplementary Fig. 4a, c), but the treatment did not change the synaptic level of vesicular glutamate—and GABA transporters (VGLUT1 and VGAT, respectively) (Supplementary Fig. 4b, d). This may imply that the increased tonic release was not due to α-syn induced changes in the quantal size of the release but due to increased basal SV recycling.

**Fig. 3:**
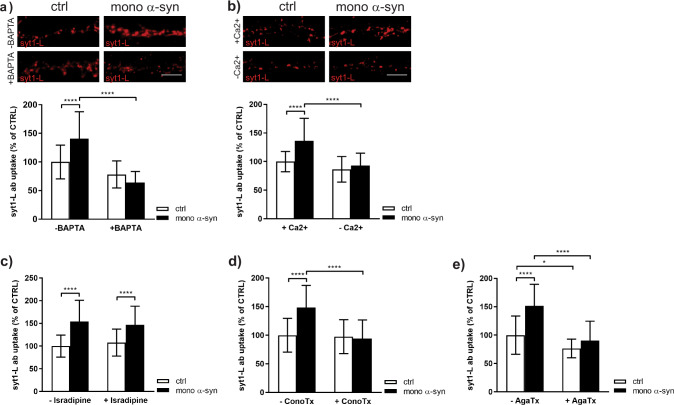
α-synuclein-induced presynaptic potentiation requires a direct Ca^2+^ influx through N-type VGCCs. **a** Representative images and statistical analysis of network-activity driven syt1L-ab uptake in primary cortical neurons treated with control solution (ctrl) or 500 nM of mono α-syn in the absence or presence of BAPTA-AM (10 µM). Data originate from three independent experiments. Number of analyzed cells: *N* = 31 ctrl; *N* = 33 mono α-syn; *N* = 29 BAPTA; *N* = 25 BAPTA/mono α-syn. Within each experiment the intensity values of treated cells were normalized to the mean intensity value of the control group (ctrl) and expressed as mean ± SD. Statistical significance was assessed using two-way ANOVA followed by Tukey’s post hoc test. Interaction *F*(1,114) = 20.59, *p* < 0.0001; mono α-syn *F* = 4.81, *p* < 0.0303; BAPTA-AM F = 66.10, *p* < 0.0001. ****p < 0.0001. Scale bar 5 µm. **b** Representative images and statistical analysis of network-activity driven syt1L-ab uptake in primary cortical neurons that are during the treatment with control solution (ctrl) or 500 nM of mono α-syn kept either in normal medium (+Ca2^+^) or in Ca2^+^-free medium (−Ca2^+^). Number of analyzed cells: *N* = 30 ctrl; *N* = 29 mono α-syn; *N* = 27 −Ca2^+^; *N* = 30 −Ca2^+^/mono α-syn from 3 independent experiments. Data are normalized to the mean intensity value of the control group (ctrl) and expressed as mean ± SD per each experiment. Two-way ANOVA followed by Tukey’s post hoc test: Interaction *F*(1,112) = 9.14, *p* < 0.0031; mono α-syn *F* = 18.88, *p* < 0.0001; −Ca2^+^ *F* = 32.86, *p* < 0.0001. ****p < 0.0001. Scale bar 5 µm. **c**–**e** Statistical analysis of network-activity driven syt1L-ab uptake in cells treated with control solution (ctrl) or 500 nM of mono α-syn in the absence or presence of VGCCs antagonists Isradipine (**c**; 1 µM), Conotoxine (**d**; ConoTx 1 µM), and Agatoxine (**e**; AgaTx 0.4 µM). All VGCCs antagonists were applied to the cells 30 min before ctrl solution or mono α-syn. Data originate from three independent experiments. Within each experimental setup, intensity values of treated cells were normalized to the mean intensity value of the control group (ctrl) and expressed as mean ± SD. Statistic was done by using two-way ANOVA followed by Tukey’s post hoc test. Number of analyzed cells: **c** *N* = 38 ctrl cells; *N* = 28 mono α-syn; *N* = 25 Isradipine; *N* = 23 Isradipine/mono α-syn; interaction *F*(1,106) = 1.20, *p* = 0.2766; mono α-syn *F* = 44.79, *p* < 0.0001; Isradipine *F* = 0.00017, *p* = 0.9896. **d** *N* = 43 ctrl cells; *N* = 24 mono α-syn; *N* = 20 ConoTx; *N* = 19 ConoTx/mono α-syn; interaction *F*(1,102) = 15.33, *p* = 0.0002; mono α-syn *F* = 11.52, *p* < 0.001; ConoTx *F* = 18.42, *p* < 0.0001. **e** *N* = 34 ctrl cells; *N* = 29 mono α-syn; *N* = 25 AgaTx; *N* = 29 AgaTx/mono α-syn; interaction *F*(1,113) = 10.06, *p* = 0.0019; mono α-syn *F* = 29.95, *p* < 0.001; AgaTx *F* = 50.20, *p* < 0.0001. *p < 0.05, ****p < 0.0001. All treatments were done at DIV 21–23.

Considering that release probability is directly proportional to the changes in intraterminal Ca^2+^ level, we also measured tonic syt1L-ab uptake in control cells and cells treated with α-syn in the presence and absence of intracellular Ca^2+^ chelator (BAPTA-AM). As seen in Fig. 3a, BAPTA completely precluded α-syn effect on presynaptic activity. The same result was achieved if the treatment with α-syn was done in the absence of extracellular Ca^2+^ (Fig. 3b). Obtained data strongly suggested that α-syn-induced facilitation of network-activity-driven presynaptic activity requires a direct influx of Ca^2+^ from the extracellular space. Considering that VGCCs are the main route for Ca^2+^ influx, we then investigated their contribution to the observed α-syn induced presynaptic augmentation. To this end, the presynaptic effect of exogenous α-syn was assessed in cells where the N-type (Cav2.2), P/Q-type (Cav2.1), and L-type (Cav1) VGCCs were pharmacologically altered. As shown, blocking L-type VGCCs with its respective blocker, Isradipine, did not exert any effect on α-syn induced rise of tonic syt1L-ab uptake, whereas both ConoTx and AgaTx (N-type and P/Q-type channel blockers, respectively) occluded α-syn-driven increase of tonic SV recycling (Fig. 3c–e). Considering that AgaTx pretreatment also impaired presynaptic activity of control cells, we may conclude that α-syn-induced increase in tonic release preferentially relies on stochastic Ca^2+^ entry through N-type Ca^2+^ channels.

### Exogenous α-synuclein exerts its presynaptic effect by altering membrane cholesterol

**Fig. 4:**
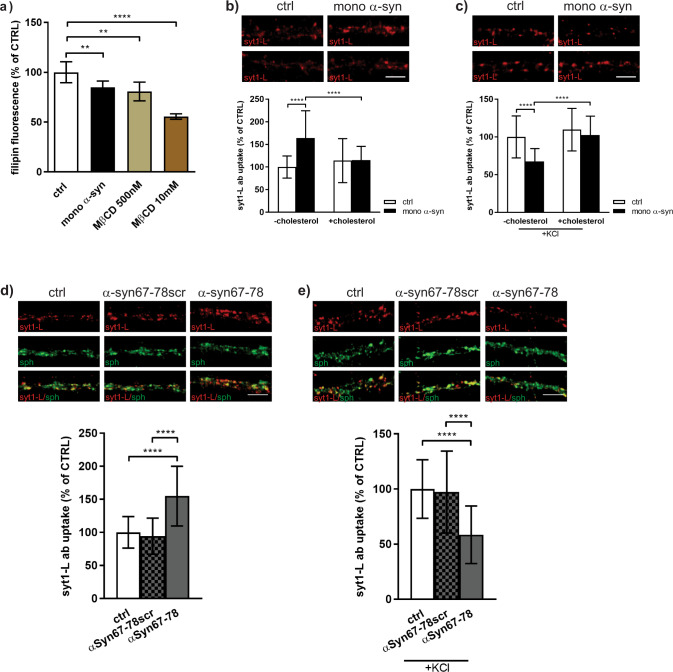
Effects of exogenous α-synuclein on presynaptic activity is cholesterol-mediated. **a)** Bar graph displaying cholesterol level determined by filipin fluorescence. Primary cortical neurons (DIV 21-23) were treated with a control solution (ctrl), 500 nM of mono α-syn, 500 nM MβCD or 10 mM MβCD for 90 min. Thereafter cells were fixed and stained with filipin. Bound filipin was extracted and the fluorescent signal was quantified using a plate reader. Data are obtained from three different experiments and within each experiment data were normalized to the respective control group. Statistical significance was determined using Kruskal–Wallis test followed by uncorrected Dunn’s test. **p < 0.01, ****p < 0.0001. **b** Representative images and statistical analysis of network-activity-driven syt1L-ab uptake in primary cortical neurons treated with control solution (ctrl) or 500 nM of mono α-syn in the absence or presence of cholesterol (1.4 mM). Data were obtained from three independent experiments. Number of analyzed cells: *N* = 32 ctrl; *N* = 34 mono α-syn; *N* = 32 cholesterol; *N* = 31 cholesterol/mono α-syn. Statistic was done using two-way ANOVA followed by Tukey’s post hoc test. Interaction *F*(1,125) = 16.83, *p* < 0.0001; mono α-syn *F* = 17.87, *p* < 0.0001; cholesterol *F* = 5.11, *p* < 0.0255. ****p < 0.0001. Scale bar 5 µm**. c** Representative images and statistical analysis of KCl-evoked syt1L-ab uptake in primary cortical neurons treated with control solution (ctrl) or 500 nM of mono α-syn in the absence or presence of cholesterol (1.4 mM). Data were obtained from three independent experiments. Number of analyzed cells: *N* = 35 ctrl; *N* = 33 mono α-syn; *N* = 34 cholesterol; *N* = 36 cholesterol/mono α-syn. Statistic was done using two-way ANOVA followed by Tukey’s post hoc test. Interaction *F*(1,134) = 8.94, *p* < 0.0001; mono α-syn *F* = 21.72, *p* < 0.0001; cholesterol *F* = 27.60, *p* < 0.0001. ****p < 0.0001. Scale bar 5 µm. **d** Network-activity-driven syt1L-ab uptake (red) in primary cortical neurons exposed for 90 min to control solution (ctrl; *N* = 52), scrambled cholesterol-binding peptide (α-syn67-78scr; *N* = 39) or cholesterol-binding peptide (α-syn67-78; *N* = 51). **e** KCl-evoked syt1L-ab uptake (red) in primary cortical neurons exposed for 90 min to control solution (ctrl; *N* = 30), scrambled cholesterol-binding peptide (α-syn67-78scr; *N* = 35), or cholesterol-binding peptide (α-syn67-78; *N* = 35). **d**, **e** Synaptophysin (sph; green) was used as a synaptic marker. Data originate from four independent experiments. Within each experiment, values in treated cells are normalized to the mean value of the control group and expressed as mean ± SD. Statistical significance was determined using Kruskal–Wallis test followed by uncorrected Dunn’s test. ****p < 0.0001. Treatments were done at DIV 21–23. Scale bar 5 µm.

The observed dual effect of α-syn on glutamate release and presynaptic activity highly resembled previously described effects of cholesterol depletion on central neurotransmission^39,40^. Several studies have already established a link between α-syn and membrane cholesterol^25,28^. Using our in vitro model, we first confirmed that similarly to α-syn treatment, cholesterol extraction by methyl-β-cyclodextrin (MβCD) led to enhanced tonic and reduced depolarization-evoked presynaptic activity (Supplementary Fig. 5a, b). Furthermore, applying a knockdown approach, we showed that increased tonic SV recycling in the presence of MβCD was also strongly dependent on Vti1a expression (Supplementary Fig. 5c). These data suggest that exogenous α-syn might reduce the level of membrane cholesterol in a similar manner to MβCD. To examine this possibility, we performed filipin staining of cells exposed either to α-syn or MβCD. As shown in Fig. 4a, quantification of the signal revealed that 90 min of the treatment with 500 nM of monomeric α-syn reduced the cholesterol level to a similar extent as the treatment with 500 nM of MβCD. In line with that, we also showed that the effect of α-syn on presynaptic activity, both network-activity driven and depolarization-evoked, could be completely reversed by cholesterol reloading (Fig. 4b, c).

Considering that α-syn contains a cholesterol-binding domain, we next hypothesized that the dual nature of α-syn-regulated SV release probability might be achieved through α-syn-cholesterol interaction. To test this, we treated our cells with α-syn peptide corresponding to its cholesterol-binding domain (aa 67–78) or scrambled control peptide. As shown on Fig. 4d, e the cholesterol binding domain of α-syn was sufficient to exert the same effect on the presynaptic activity as the full-length protein. Namely, treatment with α-syn 67-78 facilitated network-activity driven and impeded KCl-evoked syt1L-ab uptake. In vivo data, obtained by FAST measurements, also showed a strong tendency towards increased tonic and reduced evoked glutamate release upon local application of α-syn cholesterol-binding peptide (Supplementary Fig. 6a, b).

**Fig. 5:**
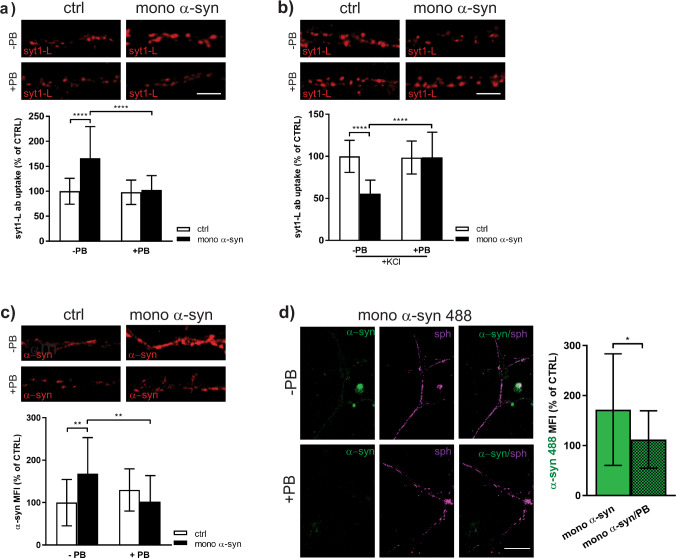
Blocking ABCA1 transporter completely occluded the effect of exogenous α-syn on SV recycling. Representative images and statistical analysis of network-activity driven (**a**) and KCl-evoked (**b**) syt1L-ab uptake in primary cortical neurons treated with control solution (ctrl) or 500 nM of mono α-syn for 90 min in the absence or presence of ABCA1 transporter inhibitor Probucol (PB; 10 µM). Data are obtained from three independent experiments and within each experiment values in treated cells are normalized to the mean value of the control group and expressed as mean ± SD. Two-way ANOVA followed by Tukey’s post hoc test. **a** *N* = 35 ctrl cells; *N* = 33 mono α-syn; *N* = 33 PB; *N* = 27 PB/mono α-syn; interaction *F*(1,124) = 19.36, *p* < 0.0001; mono α-syn *F* = 25.49, *p* < 0.0001; PB *F* = 21.89, *p* < 0.0001. **b** *N* = 29 ctrl cells; *N* = 31 mono α-syn; *N* = 28 PB; *N* = 32 PB/mono α-syn; interaction *F*(1,116) = 30.72, *p* < 0.0001; mono α-syn *F* = 30.34, *p* < 0.0001; PB *F* = 27.04, *p* < 0.0001. ****p < 0.0001. Scale bar 5 µm. **c** Representative images and statistical analysis of the level of endogenous α-syn in primary cortical neurons treated for 90 min either with ctrl solution or mono α-syn in the absence or presence of ABCA1 transporter inhibitor Probucol (PB; 10 µM). Data are obtained from three independent experiments and within each experiment values in treated cells are normalized to the mean value of the control group and expressed as mean ± SD. Two-way ANOVA followed by Tukey’s post hoc test. *N* = 25 ctrl cells; *N* = 26 mono α-syn; *N* = 28 PB; *N* = 28 PB/mono α-syn; interaction *F*(1,103) = 15.24, *p* = 0.002; mono α-syn *F* = 2.76, *p* = 0.099; PB *F* = 2.20, *p* = 0.14, **p < 0.01. Scale bar 5 µm **d** Representative images and statistical analysis of the synaptic level of α-syn 488 internalized (green) upon the treatment of primary cortical neurons for 90 min either with fluorescently labeled mono α-syn (mono α-syn 488) in the absence or presence of ABCA1 transporter inhibitor Probucol (PB; 10 µM). Synaptophysin (sph; magenta) was used as a synaptic marker. Data are obtained from three independent experiments and within each experiment values in treated cells are normalized to the mean value of the mono α-syn 488-treated group and expressed as mean ± SD. Statistic was done by using Mann–Whitney *U*-test. *p* = 0.02. *N* = 20 mono α-syn 488; *N* = 28 PB/mono α-syn 488, *p < 0.05. Treatments were done at DIV 21–23. Scale bar 20 µm.

A recent study by Hsiao et al.^41^ proposed that extracellular α-syn may act as a cholesterol acceptor that mediates cholesterol efflux from the cells and the authors demonstrated a critical role of ABCA1 transporter in this process. To determine if the aforementioned α-syn regulation of SV recycling is mediated through the ABCA1 transporter in our model system, we performed tonic and evoked syt1L-ab uptake assay in control and α-syn treated cells in the presence and absence of the ABCA1 inhibitor, probucol. Our results demonstrated that blocking the ABCA1 transporter completely occluded the effect of exogenous α-syn on both modes of SV recycling (Fig. 5a, b), confirming that α-syn modulation of presynaptic activity is, indeed, driven via cholesterol depletion. Interestingly, we also showed that blocking ABCA1 transporter prevents both upregulation of endogenous α-syn induced by 500 nM treatment and internalization of exogenous α-syn (Fig. 5c, d). This findings raised the question whether the extracellular α-syn mediated effects on presynaptic activity correlate and/or depend on how much exogenous α-syn was internalized or if these effects are primarily linked to α-syn-induced upregulation of endogenous α-syn. In order to test the later possibility we performed experiment using siRNA approach to knockdown the endogenous α-syn. Interestingly, our data showed that the effect of exogenous α-syn on SV recycling (both tonic and KCL-evoked) was hindered in the absence of endogenous α-syn. At the same time, Snca siRNA did not affect SV recycling in control cells (Supplementary Fig. 7).

### Exogenous α-synuclein regulates presynaptic activity by interfering with MAPK and Calcineurin activity

**Fig. 6:**
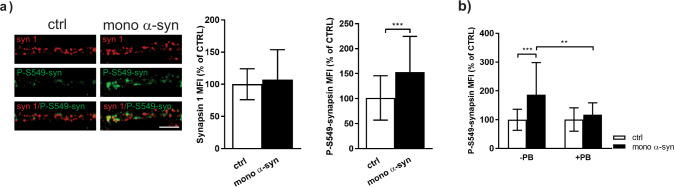
Exogenous α-synuclein regulates presynaptic activity by interfering with MAPK and Calcineurin activity. **a** Representative images and statistical analysis of synapsin 1 (syn 1, red) and P-S549-synapsin (green) in primary cortical neurons treated with control solution (ctrl) or 500 nM of mono α-syn for 90 min. Number of analyzed cells: *N* = 25 ctrl cells; *N* = 33 mono α-syn-treated cells for synapsin 1 quantification (3 independent experiments); *N* = 36 ctrl and *N* = 46 mono α-syn-treated cells for P-S549-synapsin quantification (4 independent experiments). Within each experiment, values in treated cells are normalized to the mean value of the control group and expressed as mean ± SD. Statistical significance was evaluated using Mann–Whitney *U*-test. ***p < 0.001. Scale bar 5 µm. **b** P-S549-synapsin quantification in primary cortical neurons treated with control solution (ctrl) or 500 nM of mono α-syn in the absence or presence of ABCA1 transporter inhibitor Probucol (PB; 10 µM). Two-way ANOVA followed by Tukey’s post hoc test. Number of analyzed cells: *N* = 17 ctrl; *N* = 19 mono α-syn; *N* = 18 PB; *N* = 20 PB/mono α-syn. interaction *F*(1,70) = 5.25, *p* = 0.0249; mono α-syn *F* = 11.47, *p* = 0.0012; PB *F* = 4.92, *p* = 0.0297. **p < 0.01 ***p < 0.001. Treatments were done at DIV 21–23. Scale bar 5 µm.

Changes in membrane cholesterol content are known to alter the activity of different kinases and phosphatases that are responsible for regulation of neurotransmitter release^42^. In order to determine which kinases/phosphatases might be involved in α-syn driven regulation of presynapstic activity, we quantified the synaptic level of presynaptic scaffolding protein synapsin phosphorylated at site 6 (S549) that was suggested to be primarily phosphorylated by MAPK and dephosphorylated by protein phosphatase 2B (Calcineurin (CaN))^43^. Our data revealed that treatment of cells with exogenous α-syn increased P-S549-synapsin whereas the total synapsin 1 level per individual synapse was not changed (Fig. 6a). Moreover, increased phosphorylation of synapsin was prevented if the cells were pre-treated with ABCA1 inhibitor (Fig. 6b). This observation suggested that α-syn-mediated cholesterol depletion may alter the activity of MAPK and/or CaN, although the total level of MAPK was not changed (data not shown).

## Discussion

The presence of α-syn in extracellular fluids such as interstitial fluid, cerebrospinal fluid and peripheral blood^44,45^ challenged the view that α-syn is purely an intracellular protein. It has been also well documented that both overexpressed and endogenous α-syn could be readily released from various cell types including primary neuronal cells^14^. However, the (patho)physiology of secreted α-syn is still largely unknown.

In this paper, we investigated how exogenously applied α-syn modulates synaptic structure and function, specifically focusing our study on the presynaptic compartment. Both in vivo and in vitro data firmly demonstrated that α-syn mediated regulation of presynaptic activity primarily depends on the type of SV fusion. We showed that the presence of extracellular α-syn increases network-activity-driven SV recycling and subsequent (tonic) glutamate release but reduces SV fusion and glutamate release evoked by KCl-depolarization. A number of studies supported the notion that tonic and evoked neurotransmissions are independently regulated and show spatial segregation utilizing different molecular machinery and distinct SV pools^35,46–51^. Employing syt1L-ab uptake in hypertonic solution as well as live-cell imaging technique coupled with electrical stimulation, we were able to show that exogenous α-syn does not influence the size of RRP but specifically interferes with the size of RP of vesicles. This was accompanied by dispersion of t-SNAREs (SNAP-25 and syntaxin 1a) and SNARE-associated protein complexin within individual presynaptic boutons. Both SNAP-25 and syntaxin 1, together with VAMP2/synaptobrevin are central to SV recycling and subsequent glutamate release. Genetic disruption of SNAP-25 and VAMP2/synaptobrevin was shown to be essential for evoked synaptic transmission^52,53^, whereas, the loss of syntaxin 1 completely inhibited SV fusion and priming resulting in a significant reduction of docked vesicles^54^. A conserved SNARE-binding protein, complexin, regulates both spontaneous and evoked neurotransmission. As suggested, complexin has an opposite effect on two modes of SV fusion, suppressing tonic and promoting SV release driven by depolarizing stimulus^55^. In our model system, the treatment of cells with α-syn significantly reduced the level of complexin per individual presynaptic terminal, which, together with compromised t-SNAREs, may explain global reduction of SV recycling and glutamate release evoked by KCl-depolarization. On the other hand, spontaneous neurotransmission is preferentially driven by non-canonical SNAREs, including Vti1a^37^. Utilizing KD approach, we showed that α-syn-mediated increase of tonic neurotransmission completely depends on the presence of Vti1a protein. Therefore, it seems that extracellular α-syn signals to different molecular machinery regulating the two modes of neurotransmission in an opposite manner. It is also important to mention a described interaction of α-syn with canonical SNAREs VAMP2/synaptobrevin^7^. Interestingly, non-cannonical SNAREs (synaptobrevin 7 and Vti1a) share similar SNARE and transmembrane domains to VAMP2/synaptobrevin^56^ that raises an intriguing question regarding the potential role of α-syn in their trafficking and biological function.

The dual nature of exogenously applied α-syn on presynaptic performance mirror the effect of cholesterol depletion. Membrane cholesterol keeps the balance of evoked and spontaneous neurotransmission by hindering spontaneous and sustaining depolarization-evoked SV recycling^39^. Previously it was shown that α-syn reduces the amount of plasma membrane cholesterol that dysregulates neurotransmitter release^28^. Using our model system, we corroborated α-syn mediated lowering of neuronal cholesterol and furthermore, we also demonstrated that this was indeed responsible for modulation of both network-activity-driven and depolarization-evoked SVs recycling, as both types of SV fusion could be efficiently rescued by subsequent cholesterol reloading. Taking into account that α-syn has a high affinity for cholesterol and may act as a lipid acceptor mediating cholesterol efflux through ABCA1 transporters^41^, our study further strengthened the importance of extracellular α-syn in neuronal cholesterol regulation. Namely, we showed that regulation of both modes of SV fusion in α-syn treated cells highly depends on ABCA1 activity. The literature regarding the role of ABCA1 transporters in cholesterol efflux from neuronal cells is a bit controversial. One study has shown that ABCA1 transporters do not play a role in cholesterol efflux in primary cortical neurons under physiological condition^57^, whereas two other papers reported their involvement in facilitating cholesterol efflux from both neurons and astrocytes^58,59^. However, our data clearly revealed an important role of neuronal ABCA1 in α-syn-induced presynaptic effect. Moreover, our study also reported the critical role of ABCA1 transporters in the internalization of exogenous α-syn. Existing literature has described the mechanism of endocytotic internalization for both ABCA1^60^ and α-syn^61^. Whether exogenous α-syn binds to ABCA1 transporter and gets internalized together with ABCA1 transporter has to be evaluated in the future. Also, further investigation is needed to determine if direct interaction of α-syn and ABCA1 is a prerequisite for α-syn mediated cholesterol efflux. In addition to its involvement in α-syn mediated effects on SV recycling and internalization of exogenous α-syn, blocking of ABCA1 transporters also occluded upregulation of endogenous α-syn. Therefore, the question raised was whether observed α-syn induced alterations of SV recycling depend only on the level of internalized α-syn or upregulation of endogenous α-syn plays the important role in this process. Our Snca siRNA approach revealed that endogenous α-syn plays a crucial role in extracellular α-syn-mediated effects on presynaptic activity. Obtained data could be linked to earlier described α-syn- SNARE complex interaction that promotes SNARE complex assembly^7^. At the same time, the absence of the effect of α-syn knockdown in control cells is also in a line with previously published data showing that knockout of α-syn had very little if any effect on the neurotransmitter release probably due to the functional redundancy among syn isoforms α, β, and γ^62^. In addition, our data also revealed that α-syn mediated cholesterol depletion may involve activation of different signaling molecules (e.g., MAPK and/or CaN) that impact the phosphorylation status of synapsin, one of the most important presynaptic scaffolding phosphoprotein that is involved in the regulation of neurotransmitter release through the reversible tethering of synaptic vesicles to the actin cytoskeleton^63^. Increased phosphorylation status of synapsin in α-syn treated cells might, at least partially, be responsible for increased tonic neurotransmission, whereas, KCl-induced activation of CaN might underlie decreased SV recycling in depolarized cells exposed to extracellular α-syn. However, further studies are needed to decipher the exact molecular mechanism underlying α-syn mediated regulation of presynaptic activity and glutamate release under both physiological and pathological conditions. The existence of α-syn assemblies with different structural characteristics and seeding capacities (so-called “strains”) that account for the strain-specific pathology^64^ also raises the question how those different strains may impact presynaptic structure and function.

To conclude, our study proposes that exogenous α-syn has a role in the overall maintenance of presynaptic activity. Facilitating cholesterol efflux through ABCA1 transporters and interacting with endogenous α-syn, exogenous α-syn alters plasma membrane integrity that signals to downstream signaling molecules (including MAPK, CaN, synapsin) which, in turn, mobilize molecularly distinct SV pools that give rise to increased tonic but decreased evoked glutamate release.

## Methods

### Animals

For FAST experiments, adult male C57Bl/6J mice 8 weeks of age were obtained from Charles River (Sulzfeld, Germany). Mice were kept in a 12-h light–dark cycle with ad libitum food and water. Primary cortical neurons were prepared using E18 pregnant Wistar rats obtained from Janvier Labs, France. All efforts were made to minimize the number of used animals. Animal work was done in agreement with the European Council Directive (86/609/EE) and approved by the local Animal Ethics Committee (Stockholms Norra Djurförsöksetiska Nämnd, approval number N24/12; N270/15, N269/13; 1519/2017).

### Reagents

The following reagents were purchased from Tocris, Bristol, UK: BAPTA-AM (10 µM; cat number: 2787), Isradipine (1 µM; cat number: 2004), Conotoxine (1 µM; cat number: 1085), Agatoxine (0.4 µM; cat number: 2802) and Probucol (10 µM; cat number: 2775). Methyl-β-cyclodextrin (cat. number: 128446-36-6), cholesterol (cat. number: C4951) and Filipin complex (cat. number: F9765) were obtained from Sigma-Aldrich; St. Louis, MO, USA. α-Syn cholesterol-binding peptide containing aa 67–78 (GGAVVTGVTAVA) and the corresponding scrambled control peptide (VAVAVTVGGATG) were purchased from Schafer-N Ltd. Copenhagen, Denmark. Recombinant human β-Synuclein was obtained from ENZO Biochem Inc. Farmingdale, New York (cat. number: BML-SE258-0500).

### Preparation of the recombinant α-syn monomers and oligomers

Recombinant human α-syn was expressed in *E. coli* and purified as previously described^65,66^. Briefly, α-syn plasmid vector pET11-D, containing the insert coding human α-syn, was expressed in *E. coli* BL21 (DE3) competent cells using an auto-induction method. Cells were harvested by centrifugation and treated with the osmotic shock buffer (20 mM Tris-HCl, pH-7.2, 40% sucrose), incubated for 10 min, and centrifuged again. The pellet was subsequently suspended in ice-cold deionized water, with addition of saturated MgCl_2_, and briefly incubated on ice. The periplasmic fraction of the cell lysate was collected and the majority of unwanted proteins were precipitated by acidification. The solution was fractionated on a Q-Sepharose column connected to an ÄKTA Explorer system (Cytiva). Fractions containing α-syn were identified by SDS-PAGE, pulled together and high molecular weight aggregates were removed by filtration through 30 kDa filter. The α-syn concentration was determined using NanoDrop ND1000 (Thermo Fisher Scientific, Waltham, MA, USA), protein was aliquoted, lyophilized, and stored at −20 °C. α-Syn oligomers were prepared by dissolving α-syn monomers at 10 mg/ml followed by incubation at 37 °C with 900 rpm shaking for 5 h. Insoluble material was removed and supernatant was fractioned using a Superpose 6 column (Cytiva). Oligomer fractions were collected, concentrated, and stored at 4 °C. The recombinant α-Syn was fibrillated by dissolving α-syn monomers at 1 mg/ml and incubation at 37 °C with shaking for 5 days. Obtained samples were centrifuged, obtained pellet was suspended in PBS buffer and pre-formed fibrils (PFF) were prepared by sonicating the sample to obtain a unified length of fibrils. PFFs were stored at 4 °C. Representative SDS-PAGE of used monomeric, oligomeric, and aggregated α-syn (PFF) is shown in Supplementary Fig. 8.

Preparation of α-syn488: The recombinant α-syn was marked with Alexa Fluor™ 488 NHS-ester (A20000, Thermo Fisher Scientific, Waltham, MA, USA) according to the manufacturer protocol.

### SDS-PAGE

Primary cortical neurons were maintained for 18 DIV, treated as indicated in the figure legend, and washed briefly with ice-cold washing buffer (10 mM Tris, 300 mM sucrose pH 7.4). After that cells were lysed in a buffer containing: 10 nM Tris-HCl (pH 7.4), 150 mM NaCl, 2% SDS, 1% Deoxycholate, 1% TritonX-100 and supplemented with protease and phosphatase inhibitor cocktail (Halt, Thermo Fisher Scientific). Lysates were centrifuged for 10 min at 2000 × *g* in order to eliminate cell debris. Protein concentration was determined by using BCA Protein Assay (Pierce). For SDS-PAGE, samples were mixed with a denaturing loading buffer, boiled at 95 °C for 5 min, and separated using a bis-tris acrylamide gel in an MES running buffer. For Coomassie stain, the gel was stained using Coomassie Brilliant Blue (CBB) R-250 solution. After SDS-PAGE, gels were assembled with 0.45 μm pore size PVDF membranes and the transfer was performed using The Trans-Blot Turbo Transfer System (BioRad, Hercules, CA, USA) according to manufacturer protocols. Membranes were blocked during 1 h incubation in 5% skim milk in TBST at room temperature. Next, membranes were incubated overnight at 4 °C with a primary antibodies diluted 1:1000(v/v) in 1% skim milk in TBST. Antibodies used were: anti-Vti1a (Synaptic Systems, Goettingen, Germany), anti-β-III-Tubulin (Sigma-Aldrich; St. Louis, MO, USA). Thereafter, membranes were incubated for 2 h with appropriate IRDye secondary antibodies (LI-COR, Lincoln, NE, USA) diluted 1:20000 (v/v) in 1% skim milk at room temperature and visualized in The ChemiDoc MP Imaging System (BioRad, Hercules, CA, USA). The protein ladder used was PageRuler™ Prestained Protein Ladder, 10 to 180 kDa (Thermo Fisher Scientific, Waltham, MA, USA).

### In vivo detection of glutamate release

Mice were anesthetized with isoflurane (3% for induction, 0.7–1% for maintenance) and mounted in a stereotaxic frame (David Kopf Instruments) fitted with a Cunningham mouse adapter (Stoelting Co.). Small cranial windows were drilled over the recording regions (versus bregma), prelimbic prefrontal cortex (PL): AP + 1.8; ML ± 0.3; DV −2.1 mm, and microelectrode arrays (MEAs) were inserted into this region. Glutamate dynamics were assessed on a subsecond timescale by MEA recordings with two of four electrode sites coated with L-glutamate oxidase enzyme, which breaks down L-glutamate into α-ketoglutarate and peroxide (H_2_O_2_) as previously described^67,68^. By using constant voltage amperometry with the application of a fixed potential, the H_2_O_2_ was oxidized, with electron loss, and the resulting current was recorded using a Fast Analytical Sensing Technology-16 (FAST-16 MKII) electrochemistry instrument (Quanteon). Depolarization-induced glutamate release was induced by an isotonic solution of 70 mM KCl ejected for 1 s at 1 min intervals through a glass micropipette positioned at a distance of 50–100 μm from the MEA recording sites. A MATLAB graphic interface was used to calculate concentrations of glutamate from an average of 3–5 amplitudes per mouse. The maximum amplitude of tonic and evoked glutamate (exclude amplitude less than 0.5 μM) were measured.

### Primary cortical neurons

Primary cortical neurons were prepared as described previously^69^. Briefly, E18 embryos were decapitated and cortical tissue was further dissociated using neuronal isolation enzyme with papain (Thermo Fisher Scientific, Waltham, MA, USA; cat. number: 88285). For immunocytochemistry and synaptotagmin 1 antibody uptake assay, cells were plated on poly-D-lysine-coated glass coverslips (round 12 mm, A. Hartenstein GmbH) at a density of 50,000 cells/coverslip and for synaptotagmin-1-cypHer imaging on 18 mm round glass coverslip at a density of 100.000 cells/coverslip. For filipin staining cells were plated in 6- or 12-well plate at a density of 300,000 cells/well and 100,000 cells/well, respectively. Cells were initially plated in DMEM (Thermo Fisher Scientific, Waltham, MA, USA; cat. number: 0938025) supplemented with 10% FBS (Thermo Fisher Scientific, Waltham, MA, USA; cat. number: 10500064), 0.8 mM glutamine Thermo Fisher Scientific, Waltham, MA, USA; cat. number: 35050038) and penicillin/streptomycin (100 U/ml penicillin, 100 μg/ml streptomycin) (P/S; Thermo Fisher Scientific, Waltham, MA, USA; cat. number: 15140122). 24 hours later DMEM was replaced with Neurobasal medium (Thermo Fisher Scientific, Waltham, MA, USA; cat. number: 21103049) supplemented with B-27 50× (20 ml/l) supplement (Thermo Fisher Scientific, Waltham, MA, USA; cat. number: 17504044), 0.8 mM glutamine and P/S (100 U/ml penicillin, 100 μg/ml streptomycin). Cells were kept in a humidified incubator with 5% CO_2_ for up to 3 weeks and fed every third day. All treatments were performed between DIV 21–23, except siRNA that was done on DIV18 for 96 h.

### Functional imaging and Immunocytochemistry

For syt1L-ab uptake assay, primary cortical neurons were plated on poly-D-lysine coated coverslips (round 12 mm) in 24 well plates at the density of 50.000 cells per coverslip. After the appropriate treatment, cells were washed with freshly prepared Tyrode’s buffer (containing in mM: 119 NaCl, 2.5 KCl, 2 CaCl_2_, 2 MgCl_2_, 30 glucose, and 25 Hepes; pH 7.4). For network activity driven synaptotagmin 1 antibody uptake assay (syt1L-ab uptake), fluorescently labeled anti-Synaptotagmin1 lumenal domain antibody (1:250; Synaptic Systems; cat. number: 105311C3) was diluted in Tyrode’s buffer and applied on cells for 20 min at 37 °C. For depolarization-evoked syt1L-ab uptake, the antibody was diluted in Tyrode’s buffer with high KCl (50 mM) and incubated with the cells for 4 min at 37 °C and to assess the size of RRP, syt1L-ab uptake was done in hypertonic solution (Tyrode’s buffer containing 500 mM sucrose). After the three steps of washing, cells were fixed with 4% paraformaldehyde for 3 min and then blocked/permeabilized with 10% FBS/0.3% TritonX-100/0.1% Glycine in PBS for 30 min at room temperature. Primary antibodies diluted in 3% FBS/PBS were applied over night at 4 °C. The following primary antibodies were used: guinea pig anti-Synaptophysin 1 (1:1000; Synaptic Systems, Göttingen, Germany; cat. number. 101 004); mouse anti-SNAP25 (1:1000; Synaptic Systems, Göttingen, Germany; cat. number. 111 011); mouse anti-Synaptobrevin2/VAMP2 (1:1000; Synaptic Systems, Göttingen, Germany; cat. number 104 211); mouse anti-Synapsin 1 (1:1000; Synaptic Systems, Göttingen, Germany; cat. number 106 011); rabbit anti-synatxin 1 (1:1000; Synaptic Systems, Göttingen, Germany; cat. number 110 302); rabbit anti-complexin 1/2 (1:1000; Synaptic Systems, Göttingen, Germany; cat. number 122 002); rabbit anti-Piccolo (Aczonin) (1:1000; Synaptic Systems, Göttingen, Germany; cat. number 142 003); rabbit anti-Bassoon (1:1000; Synaptic Systems, Göttingen, Germany; cat. number 141 003); rabbit anti-RIM 1/2 (1:1000; Synaptic Systems, Göttingen, Germany; cat. number 140 203); rabbit anti-Homer 1 (1:1000; Synaptic Systems, Göttingen, Germany; cat. number 160 003); rabbit anti-Phospho-S549-Synapsin 1 (1:500; PhosphoSolution, Aurora, CO, USA; cat. number P1560-549); rabbit anti-α-synuclein (C-20) (1:500; Santa Cruz Biotechnology, Dallas, TX, USA; cat. number Sc-7011-R); rabbit anti-Vti1a (1:500; Synaptic Systems, Göttingen, Germany; cat. number 165 003); rabbit anti-VGLUT 1 (1:1000; Synaptic Systems, Göttingen, Germany; cat. number 135 302); rabbit anti-VGAT (1:1000; Synaptic Systems, Göttingen, Germany; cat. number 131 002). Secondary antibodies diluted also in 3% FBS were applied during 1 h at RT: Anti-rabbit Alexa Fluor 488 (cat. number A-11008); Anti-mouse Alexa Fluor 568 (cat. number A-11031); Anti-guinea pig Alexa Fluor 647 (cat. number A-21450). All were used in the dilution of 1:1000 (Thermo Fisher, Waltham, MA, USA). Coverslips were mounted on microscopics slides using Mowiol 4-88 (Roth; cat. number 9002-89-5). For each condition, two coverslips were processed in parallel.

For Synaptotagmin-1-cypHer imaging primary cortical neurons were plated on poly-D-lysine coated coverslips (round 18 mm) in 12 well plates at the density of 100.000 cells per coverslip. Synaptotagmin-1-cypHer imaging was essentially done as described in^70^ using anti-Synaptotagmin1 lumenal domain antibody (1:100; Synaptic Systems; cat. number: 105311CpH). Cells were incubated with this antibody diluted in modified Tyrode’s buffer (120 mM NaCl, 5 mM KCl, 2 mM MgCl_2_, 2 mM CaCl_2_, 10 mM glucose, and 18 mM NaHCO_3_, pH 7.4) for 3 hours at 37 °C on order to label all active synapses. Live imaging was performed using an inverted microscope (Observer. D1; Zeiss) equipped with an EMCCD camera (Evolve 512; Photometrics) controlled by VisiView (Visitron Systems GmbH) software, using 63x objective and Cy5 ET filter set (exciter 620/60, emitter 700/75, dichroic 660 LP) (Chroma Technology Corp.). 18 mm coverslips were placed in the imaging chamber supplemented with platinum wire electrodes, at 26 °C in the presence of Bafilomicyn A1 (1 µM; Calbiochem; cat. number:196000). A stream of images was acquired at 10 Hz. The action potentials were evoked by delivering 1 ms constant voltage pulses at 20 Hz using S48 stimulator (GRASS Technologies). Stimulation protocol of 40 AP at 20 Hz was applied to release vesicles from the RRP. Upon 2 min without stimulation, a train of 200 AP at 20 Hz was applied to release vesicles from RP. Active synapses were identified upon subtracting the average of 10 frames directly after the onset of stimulation from the average of the first 10 frames of the baseline (before stimulation). The mean immunofluorescent (IF) intensities were measured in ROIs with a radius of 1.87 μm, centered over each responding synapse using a custom-made macro in ImageJ (NIH, http://rsb.info.nih.gov/ij/). The final fluorescence traces were corrected for the bleaching factor that was estimated from the bleaching of the naïve puncta on the same image.

### siRNA transfection to reduce Vti1a and α-syn expression in primary neurons

siRNA transfections were done according to manufacturer’s protocol using either SMARTpool Accell rat Vti1a (65277) (Dharmacon, cat. number: E-099855-00-0010) or α-syn Accell rat Snca (Dharmacon, cat. number: E-090827-00-0005). For the control experiment, cells were treated with Accell Non-targeting Pool siRNA (cat. number: D-001910-10-05). siRNA (final concentration 1 µM) was applied to cells for 96 h before the treatment with either control solution or α-syn. For each condition 2 coverslips were processed at the same time. siRNA treatment was done on primary neurons at DIV 18.

### Visualization of membrane cholesterol by using filipin staining

After the treatment, primary cortical neurons were fixed with 4% paraformaldehyde for 5 min, washed with PBS, and incubated for 1 h in PBS containing 1% Triton X-100. Thereafter, cells were incubated for 1 h with filipin (100 µg/ml) diluted in 10% FBS/PBS and then 30 min in 1% SDS. All steps were done at room temperature. Finally, the fluorescence (ex 360 nm/em 470 nm) was measured in the solution using a Tecan plate reader (Tecan Life Science, Männedorf, Switzerland).

### Microscopy, image analysis, and statistics

Image acquisition was done on ZEISS LSM 880 Airyscan confocal laser scanning microscopy equipped with ZEN2.1 software, using Plan-Apochromat ×63/1.4 Oil DIC M27 63x oil objective. For quantifications, the same settings were used for all coverslips quantified within one experiment. The signal was quantified using ImageJ (https://imagej.nih.gov/ij/docs/faqs.html) and OpenView software^71^. Background subtraction was done in ImageJ. Synaptic puncta were defined along 20 µm of proximal dendrite by setting a rectangular region of interest (ROI) in channel positive for Synaptophysin (chosen as synaptic marker) by using OpenView software. Mean immunofluorescence intensities were measured in the synaptic ROIs in all channels. Within each independent experiment, data were normalized to the mean of the control group and expressed as mean ± SD. For the presentation images were processed by using ImageJ and PhotoShop (Adobe Systems).

Statistical analyses were done using GraphPad Prism (GraphPad, San Diego, CA, USA). The chosen statistical tests, number of used animals, and number of cells analyzed per each group are indicated in figure legends. D’Agostino & Pearson normality test was used to check the normality distribution of the data and, accordingly, parametric or nonparametric tests were applied. Statistical significance was assessed as *p < 0.05; **p < 0.01; ***p < 0.001; ****p < 0.0001.

## Supplementary information

Supplementary Information

## Data Availability

All data and materials are available upon request to the authors.
