# Supplementary material for: α-Synuclein induced cholesterol lowering increases tonic and reduces depolarization-evoked synaptic vesicle recycling and glutamate release
Source: NPJ Parkinsons Dis. 2022 Jun 7;8:71. doi: 10.1038/s41531-022-00334-7 (PMC9174203; doi:10.1038/s41531-022-00334-7)
Supplement: Supplementary file 1 — Supplementary Information [file 41531_2022_334_MOESM1_ESM.pdf]

Supplementary figures and legends

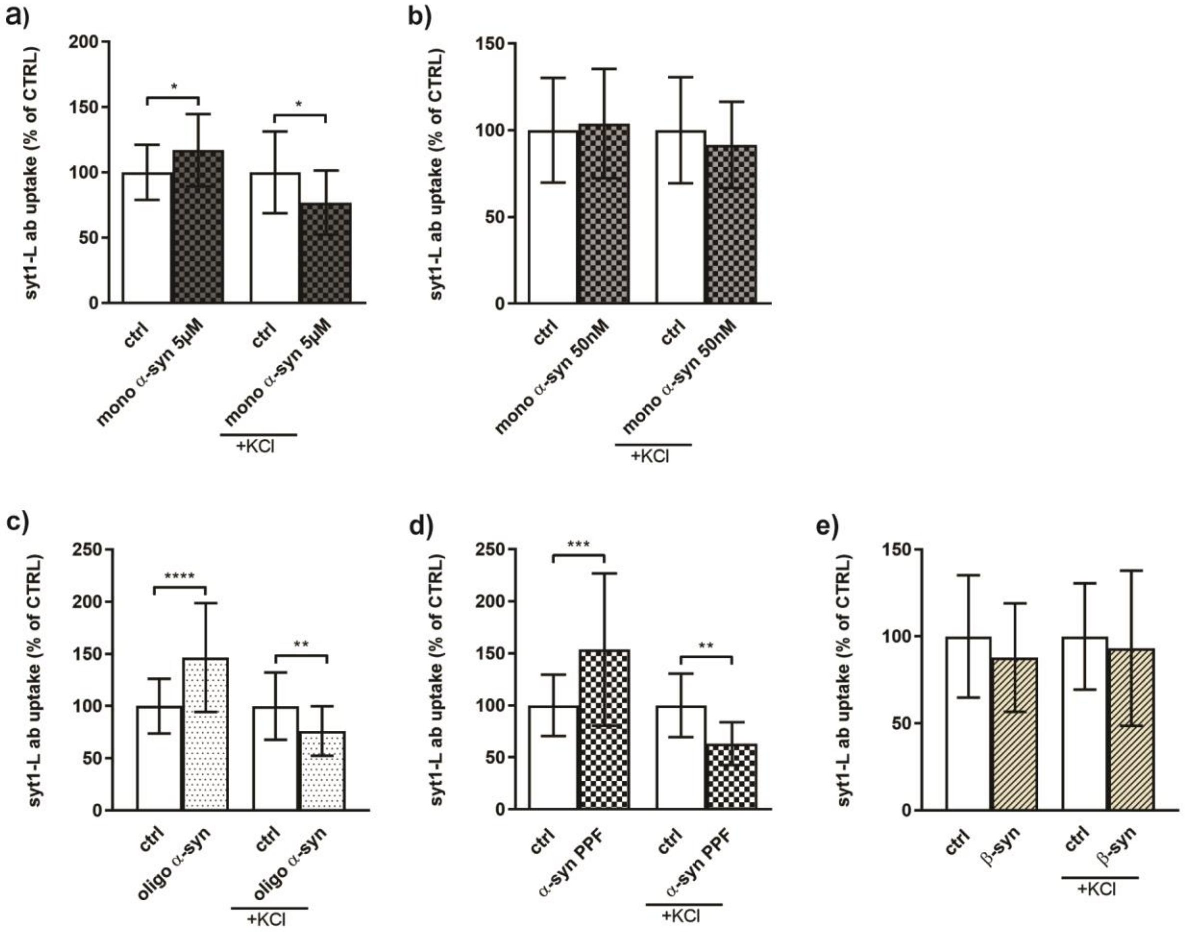

Supplementary figure 1. Oligomeric and aggregated α-syn alters SV recycling.

Quantification of network activity driven and KCL-evoked syt1-L ab uptake in control cells and cells treated with 5 µM **(a)** and 50 nM **(b)** of mono α-syn. Number of analysed cells (a): ctrl N=24, 5 µM mono α-syn N=31; (b) ctrl N=15, 50 nM mono α-syn N=19. Statistics was done with Student's t-test. $p<0.05$. **c)** Graph represents statistical analysis of both network-activity driven and KCl-evoked syt1-L ab uptake in primary cortical neurons treated with control solution or 500 nM of oligomeric α-syn. For network-activity driven syt1-L ab uptake number of analyse cells was N=44 for ctrl cells and N=44 for oligo α-syn treated cells. Data originate from 3 independent experiments. Statistics was done using Mann-Whitney U test. ****$p<0.0001$. For guantification of KCl-evoked syt1-L ab uptake in primary neurons, the number of analysed cells was N=24 cells (ctrl) and N=23 (oligo α-syn). Data are pooled from 2 experiments, normalized

to the mean value of the control group and expressed as mean ± SD per each experiment. Statistic assessed by using Mann-Whitney U test. **p<0.01. **d)** Graph represents statistical analysis of both network-activity driven (N=30 ctrl cells; N=29 PFF treated cells) and KCl-evoked syt1-L ab uptake (N=11 ctrl cells; N=8 PFF treated cells) in primary cortical neurons treated for 90 min with control solution or 500 nM of α-syn PFF. Statistical assessment was done by using Mann-Whitney U test for network-activity driven and Student's t-test for KCl-evoked release. **e)** network-activity driven (N=25 ctrl cells and N=26 β-syn treated cells; Student's t-test) and KCl-evoked (N=23 ctrl cells and N=30 β-syn treated cells; Mann-Whitney U test) syt1-L ab uptake in primary cortical neurons treated for 90 min with control solution or 500 nM of β-syn. Through all data setes values in treated cells are normalized to the mean value of the control group and expressed as mean ± SD.

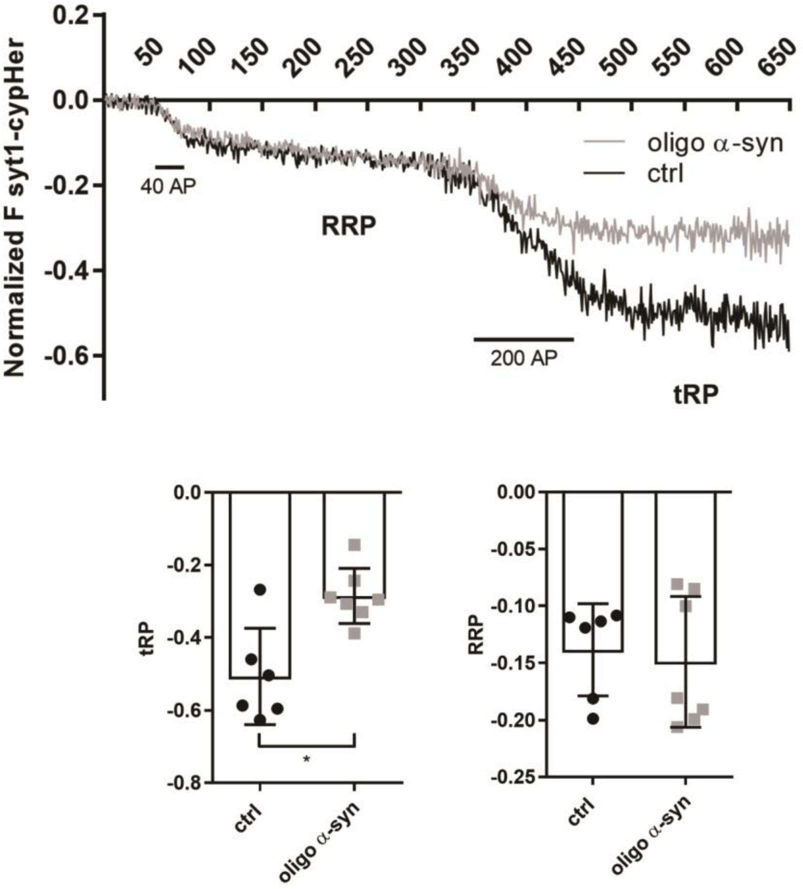

Supplementary figure 2. Oligomeric α-syn reduced the size of recycling pool without altering readily releasable pool of SVs.

Average syt1-cypHer fluorescence traces and corresponding quantifications reporting SV pool sizes from control (ctrl) and oligo α-syn (500 nM; 90 min) treated primary neurons. Stimulation protocol of 40AP at 20 Hz was applied to release vesicles from the RRP. Upon 2 min without stimulation a train of 200 AP at 20 Hz was applied to release vesicles from RP (representing the tRP). Fluorescence responses corresponding to the size of RRP and RP are given as fractions of the total Syt1-L-CypHer labeling. Data originate from N=6 ctrl cells and N=7 oligo α-syn treated cells. Statistic was done using Mann-Whitney U test. $*p<0.05$.

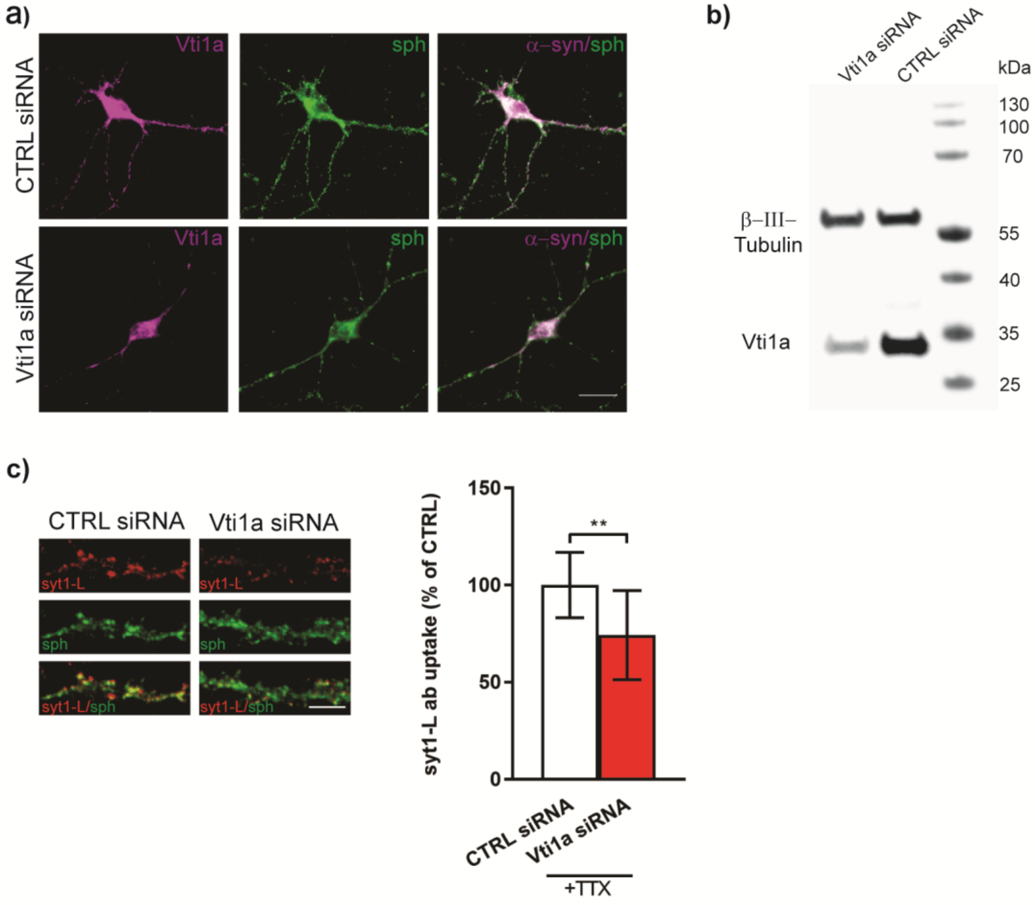

Supplementary figure 3. Knockdown of Vti1a attenuates AP-dependent presynaptic activity.

a) Representative images of Vti1a (magenta) and synaptophysine (sph, green) immunofluorescence in primary cortical neurons upon 96 h of incubation with CTRLsiRNA or Vti1a siRNA. Scale bar 20 µm**. b)** Representative WB showing the efficacy of Vti1a siRNA. Primary cortical neurons (DIV 18) were exposed either to CTRL siRNA or Vti1a siRNA for 96h. Cells were lysed and equal amount of proteins were loaded on the gel. β-III- tubulin was used as internal loading control. **c)** Representative images and statistical analysis of syt1-L ab uptake (red) in the presence of TTX (1 µM) in primary cortical neurons exposed to CTRLsiRNA (N=12 cells) or Vti1a siRNA (N=11 cells) for 96 h. Synaptophysine (sph; green) was used as synaptic marker. Statistical significance was assesses by using Student's t-test. **p<0.01. Scale bar 5 µm.

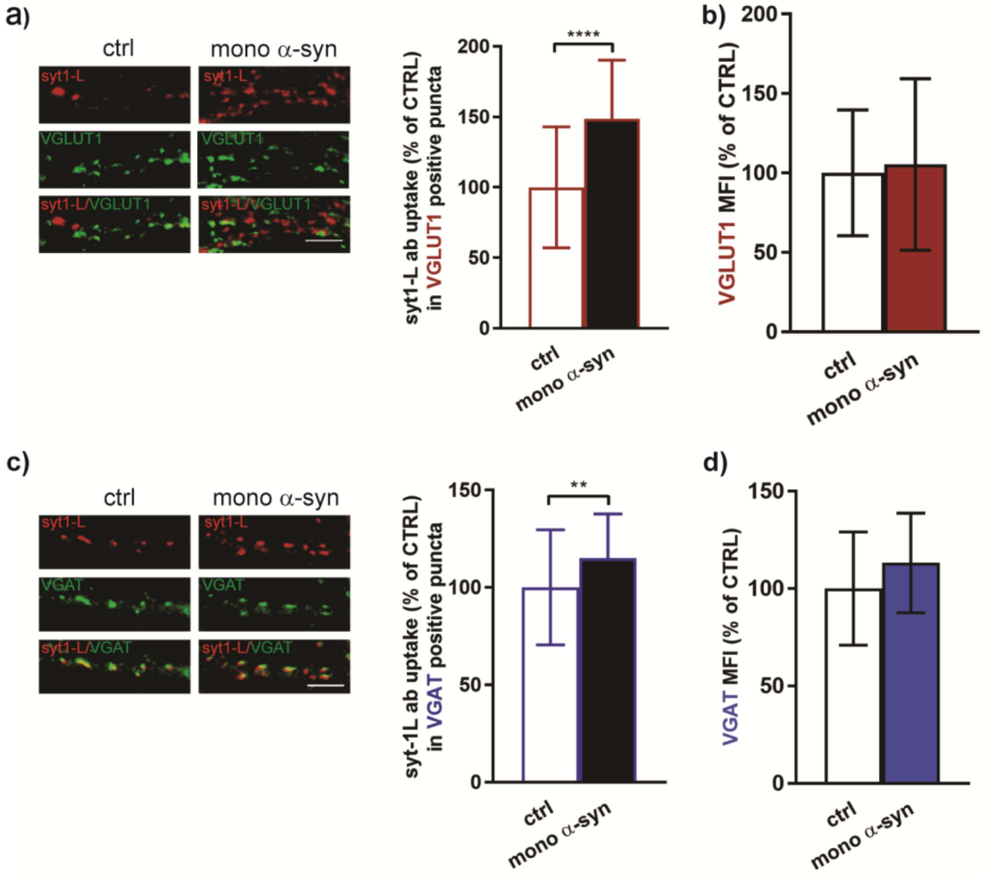

Supplementary figure 4. Exogenous α-synuclein alters the presynaptic activity in both excitatory and inhibitory synapses.

a) Representative images and corresponding statistic of network-activity driven syt1-L ab uptake (red) in primary cortical neurons treated for 90 min with control solution (ctrl; N=37) or 500 nM mono α-syn (N=31). Data originate from 3 independent experiments. The signal of syt1-L ab uptake was measured in VGLUT1 positive puncta (green). Statistic was done using Mann-Whitney U test. ****$p<0.0001$. **b)** Quantification of VGLUT1 immunofluorescence shown in (a) along 20 µm of proximal dendrite. Data originate from 4 independent experiments. Number of analyzed cells N=47 ctrl and N=36 mono α-syn. Mann-Whitney U test. **c)** Representative images and corresponding statistic of network-activity driven syt1-L ab uptake (red) in primary cortical neurons treated for 90 min with control solution (ctrl; N=32) or 500 nM mono α-syn (N=28). Data originate from 3 independent experiments. The signal of syt1-L ab uptake was measured in VGAT positive puncta (green). Statistic was done using Mann-Whitney U test. **$p<0.01$. **d)** Quantification of VGAT immunofluorescence shown in (c) along 20 µm of proximal dendrite. Data

originate from 3 independent experiments. Number of analyzed cells N=39 ctrl and N=28 mono α-syn. Statistic was done using Student's t-test. In all experiments values in treated group are normalized to the mean value of the control group and expressed as mean ± SD. Scale bar 5 µm.

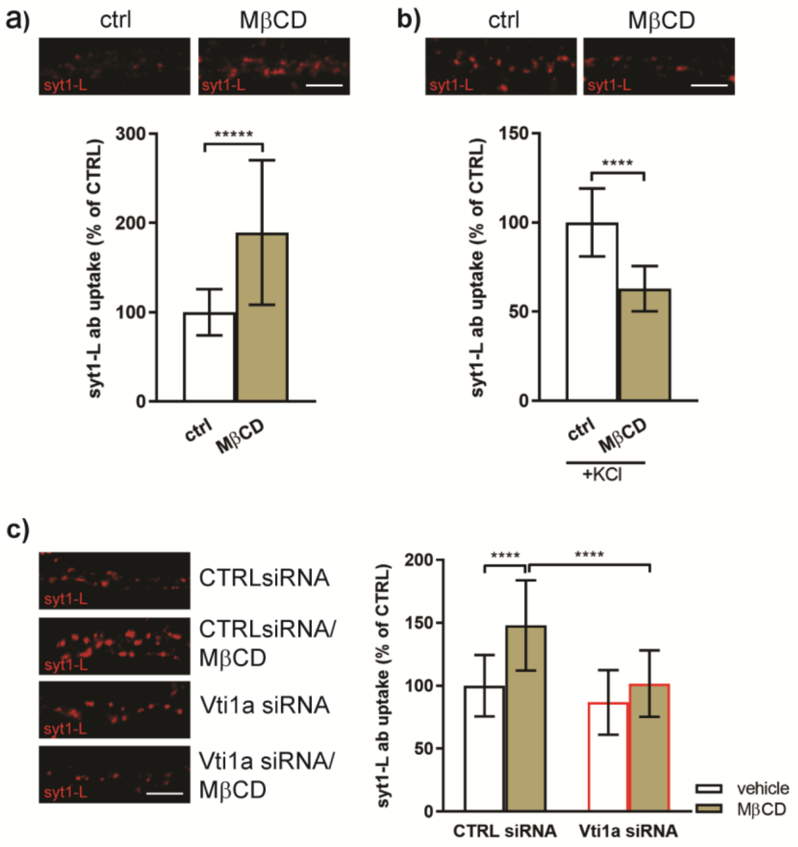

Supplementary figure 5. Cholesterol depletion enhances tonic and reduces depolarization-evoked presynaptic activity.

a) Network-activity driven syt1-L ab uptake in control cells (ctrl; N=30) and cells treated for 90 min with 500 nM MβCD (N=28). Statistic was done using Mann-Whitney U test. ****p<0.0001. **b)** KCl-evoked syt1-L ab uptake in control cells (ctrl; N=29) and cells treated for 90 min with 500 nM MβCD (N=28). Student's t-test. ****p<0.0001. **c)** Representative images and statistic of network-activity driven syt1-L ab uptake in cells exposed to CTRLsiRNA or Vti1a siRNA for 96 h without and with subsequent treatment with 500 nM MβCD. Number of analyzed cells: N=33 CTRLsiRNA; N=31 CTRLsiRNA/ MβCD; N=30 Vti1a siRNA; N=32 Vti1a siRNA/ MβCD. Statistical significance was assesses by using two-way ANOVA followed by Tukey's post hoc test, interaction F(1,122)=10.58, p=0.0015; MβCD F=38.40, p<0.0001; Vti1a siRNA F=34.40, p<0.0001. All data originate from 3 independent experiments. In each experiment values in treated group are normalized to the mean value of the control group and expressed as mean ± SD. Scale bar 5 µm.

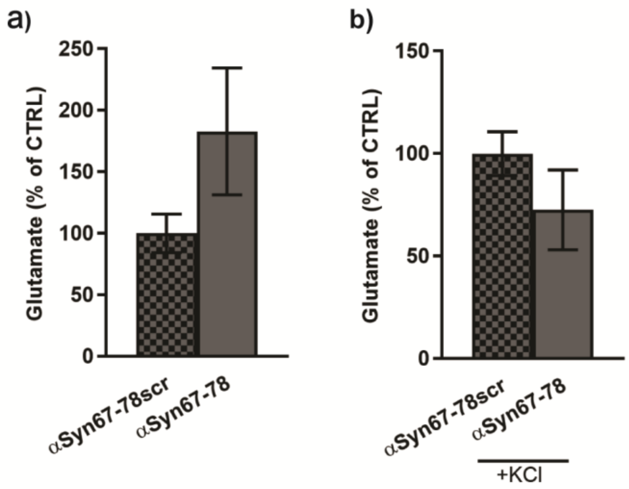

Supplementary figure 6. Glutamate release measured upon local application of α-syn 67-78 into prelimbic region of prefrontal cortex.

Quantification of tonic **(a)** and KCl-evoked **(b)** glutamate release measured in the prelimbic region of prefrontal cortex in mice upon local application of α-syn67-78scr (N=11) or 500 nM of α-syn67-78 (N=11). Statistical significance was assessed using Mann-Whitney U test (a) or Student's t-test (b).

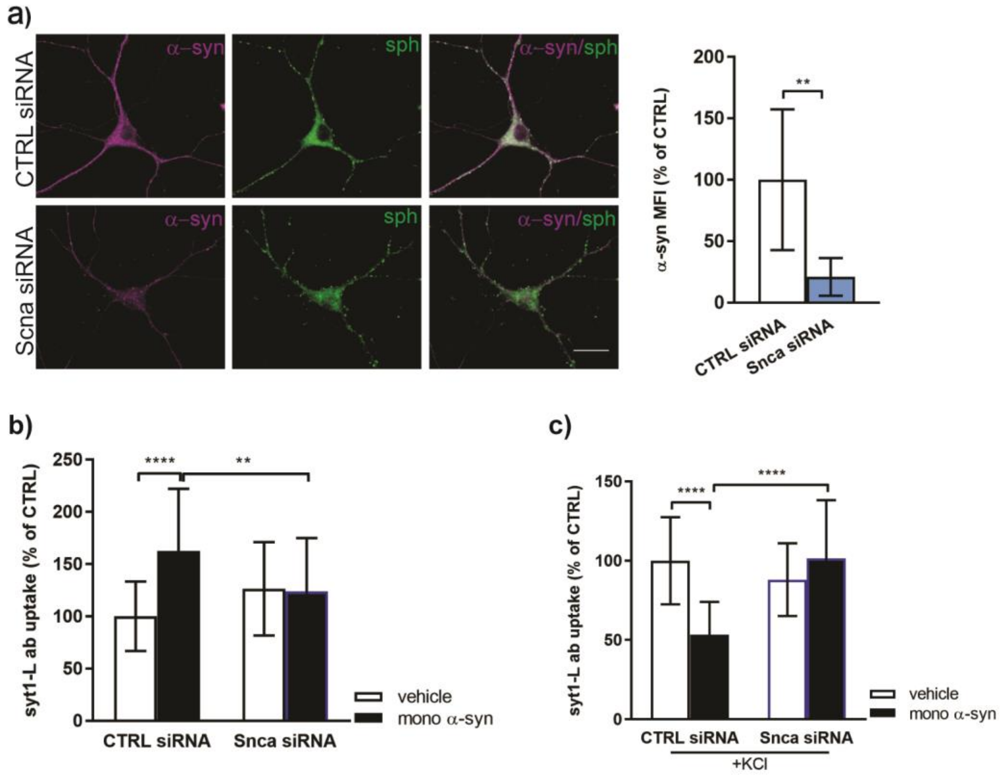

Supplementary figure 7. Knockdown of endogenous α-syn occluded the effect of exogenous α-syn on SV recycling.

a) Representative images and corresponding quatigication of α-syn (magenta) and synaptophysin (sph, green) immunofluorescence in primary cortical neurons upon 96 h of incubation with CTRL siRNA or Snca siRNA. Scale bar 20 µm**.** N=13 CTRL siRNA, N=8 Snca siRNA cells. Statistical significance was assessed by using Student's t-test. **$p<0.01$. **b)** Quantification of network-activity driven syt1-L ab uptake in cells exposed to CTRLsiRNA or Snca siRNA for 96 h. Number of analyzed cells: N=31 CTRLsiRNA; N=35 CTRLsiRNA/mono α-syn (500 nM; 90 min), N=29 Snca siRNA, N=33 Snca siRNA/mono α-syn (500 nM; 90 min). Statistical significance was assesses by using two-way ANOVA followed by Tukey's post hoc test, interaction $F(1,124)=14.34$, $p=0.0002$; α-syn $F=12.23$ $p<0.0007$; Snca siRNA $F=0.54$, $p=0.464$. Data originate from 3 independent experiments**. c)** Quantification of KCl-evoked syt1-L ab uptake in cells exposed to CTRLsiRNA or Snca siRNA for 96 h. Number of analyzed cells: N=16 CTRLsiRNA; N=21 CTRLsiRNA/mono α-syn (500 nM; 90 min), N=21 Snca siRNA, N=23 Snca siRNA/mono α-syn (500 nM; 90 min). Statistical significance was assesses by using two-way ANOVA followed by Tukey's post hoc test, interaction $F(1,77)=23.09$, $p<0.0001$; α-syn $F=7.00$ $p=0.009$; Snca siRNA $F=8.39$, $p=0.0049$. Data originate from 3

independent experiments. In all experiments values in treated group are normalized to the mean value of the control group and expressed as mean ± SD.

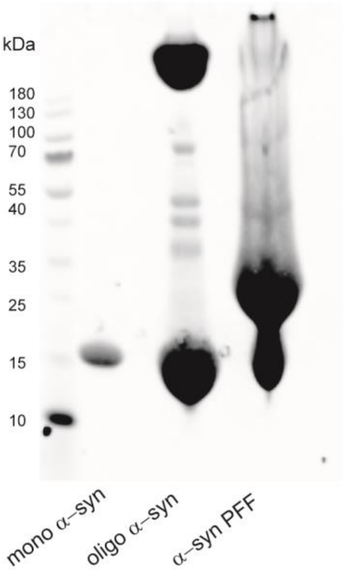

Supplementary figure 8. Representative WB of monomeric, oligomeric and α-syn PFF

Scanned SDS-PAGE gel showing α-syn monomer, oligomer and PPF samples.
